# INSIGHT-2: mechanistic studies into pregnancy complications and their impact on maternal and child health—study protocol

**DOI:** 10.1186/s12978-024-01911-0

**Published:** 2024-11-28

**Authors:** Carlotta Valensin, Emilie J. M. Côté, Daniela Pereira-Carvalho, Rachael A. Gardner, Glen Nishku, Caitlin L. Giles, Carolyn Gill, Anna Brockbank, Lisa Story, Andrew H. Shennan, Natalie Suff, Deena L. Gibbons, Rachel M. Tribe

**Affiliations:** 1 Department of Women and Children’s Health, St Thomas’ Hospital, King’s College London, London, UK; 2 Department of Perinatal Imaging, St Thomas’ Hospital, King’s College London, London, UK; 3 Guy’s and St Thomas’ National Health Service Foundation Trust, London, UK; 4 Peter Gorer Department of Immunobiology, Guy’s Hospital, King’s College London, London, UK

**Keywords:** Pregnancy complications, Pregnancy exposures, Observational study, Study cohort, Preterm birth, Infancy, Type 1 diabetes, Islet autoimmunity, Infant health

## Abstract

**Background:**

Pregnancy and early childhood cohorts provide a framework for investigating the complex interplay between early-life exposures and health outcomes, thereby informing prevention strategies and interventions to improve maternal and child health. In this paper, we outline the objectives, methodologies and expected contributions of INSIGHT-2, a comprehensive cohort study dedicated to advancing our understanding of pregnancy and pregnancy complications towards improving the health and well-being of mothers and their offspring.

**Methods:**

Over the course of 5 years, the study aims to establish a diverse cohort of 1700 pregnant women and to follow up their children up to 2 years of age. Recruitment targets participants with healthy pregnancies, preexisting conditions, and/or risk factors for pregnancy complications or later child health problems. Clinical and lifestyle data and a range of biological samples will be collected, providing a comprehensive resource for biomarker investigations and cross-sectional analyses. It is anticipated that the cohort will continue beyond this initial 5-year plan.

**Discussion:**

By gathering a wide range of biological samples and using diverse analytical techniques, this study supports broad participation, potential replication and collaboration across various sites. The extensive collection of longitudinal data and samples not only facilitates current investigations but also establishes a biobank for future research. The exploration of pre-pregnancy and pregnancy factors that may contribute to disease processes and impact fetal well-being and future health will provide a comprehensive picture of disease mechanisms in both mothers and children, facilitating the identification of biomarkers for the prediction, diagnosis, and management of pregnancy complications. Additionally, our diverse population allows for the capture of various pregnancy complications and outcomes, enhancing external validity and addressing health disparities. This comprehensive design ultimately aims to improve maternal and child health outcomes by providing a valuable longitudinal study of the relationships among the in utero environment, pregnancy management, and long-term maternal and child health, ensuring that findings are relevant and beneficial to a broader population.

**Supplementary Information:**

The online version contains supplementary material available at 10.1186/s12978-024-01911-0.

## Background

Every expectant parent anticipates a healthy pregnancy and baby. However, many of pregnancies in the UK are affected by complications such as spontaneous preterm birth (sPTB, 3–6% [1, 2]), pre-eclampsia (PE) (< 2% [3]), and gestational diabetes mellitus (GDM, 8–24% [4]). The rates of these complications are even higher in some regions of the world and in women with preexisting medical conditions. Furthermore, they not only pose immediate risks but are also linked to long-term health issues for both mothers and children [5–8], including an increased likelihood of developing chronic conditions [9].

To better understand and develop interventions for these complications, cohorts have been established that follow women and children during pregnancy and after birth. While many studies have taken an epidemiological approach (e.g., [2, 10, 11]), others have conducted biological sampling [12–16] to allow for more detailed phenotyping [17–21]. Examples include our previous studies, CLIC and INSIGHT. Both provide a biobank and a clinical database, with CLIC focusing on identifying biomarkers for the early prediction of preterm labour [22–24] and INSIGHT focusing on biomarkers for risk prediction of sPTB [19, 25–28]. This combined approach is critical for elucidating the pathophysiological mechanisms of pregnancy complications and identifying biomarkers for risk stratification, diagnosis, and clinical management [29].

To provide data and biological samples that support investigations into these issues, we established a new maternal‒child cohort, “INSIGHT-2: Mechanistic Studies into Pregnancy Complications and their Impact on Maternal and Child Health”. Cosponsored by King's College London (KCL) and Guy's and St Thomas' NHS Foundation Trust (GSTT), INSIGHT-2 is a longitudinal pregnancy and infancy cohort follow-up study designed to investigate the biological mechanisms driving pregnancy complications and evaluate their impact on the future health of both participants and their offspring.

The unique strength of INSIGHT-2 lies in the comprehensive linkage between mothers and children. This allows for the elucidation of the long-lasting impact of prenatal exposure on the health outcomes of offspring, as exemplified by the heightened risk of type 1 diabetes mellitus (T1DM) among babies born prematurely [30, 31]. Furthermore, many pregnancy complications often cooccur, such as GDM and PE [32], emphasising the need for a comprehensive and diverse cohort. The latter will enable us not only to discern findings across various demographic groups but also to develop tools and interventions that can benefit all.

In the long term, enhancing our understanding of the mechanisms behind healthy pregnancies and identifying biomarkers for complications holds promise for improving clinical care. This research could support the development of earlier and more accurate prediction methods, facilitating targeted interventions that may help mitigate risks for both mothers and children. Over time, this could inform more tailored approaches to address individual needs. By capturing a comprehensive and diverse set of data, INSIGHT-2 aims to contribute valuable insights that may guide future preventative and therapeutic strategies.

## Methods

### Study objective

**Fig. 1:**
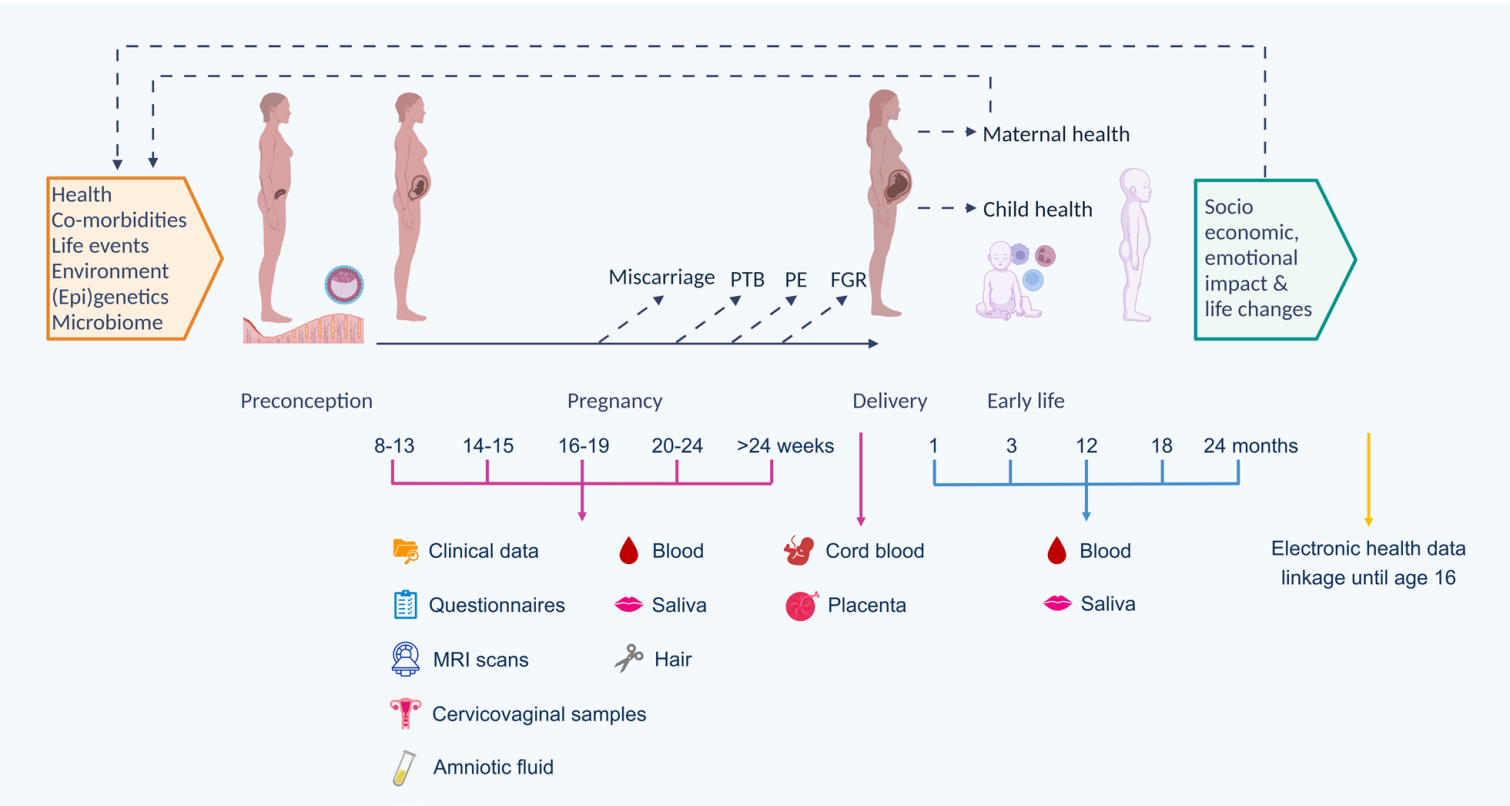
INSIGHT-2 cohort structure and participation timeline. PTB: preterm birth, PE: preeclampsia, FGR: fetal growth restriction, MRI: magnetic resonance imaging. Created in BioRender. Tribe, R. (2023). BioRender.com/b99l429

Our objective is to establish a longitudinal pregnancy and early childhood cohort providing clinical data and biological samples to support studies elucidating the impact of pre-pregnancy and pregnancy factors on maternal, fetal, and child health outcomes (Fig. 1).

INSIGHT-2 seeks to recruit diverse pregnancy profiles, including the following:

- Participants with healthy pregnancies.
- Participants with risk factors for pregnancy complications, including GDM and sPTB.
- Participants with preexisting conditions such as systemic lupus erythematosus (SLE) and T1DM or with first-degree relatives/partners with these conditions.

### Study size and population

Our target cohort size is 1700 participants. Recruitment began in November 2023 and is expected to continue for five years, with our recruitment centres being general and disease-specific antenatal clinics, specialist clinics for PTB, and maternal medicine surveillance. Demographics based on our previous INSIGHT study and our 230 participants enrolled up to July 2024 show that recruitment reflects the local population of the Lambeth area in terms of ethnicity (Table 1).

**Table 1:** Ethnicity of the population

Ethnicity^a^	Population			
	England and Wales 2021 [33]	Lambeth 2022 [34]	INSIGHT study Nov. 2016–Oct. 2023	INSIGHT-2 study Nov. 2023–Jul. 2024
Asian or Asian British	9%	6%	10%	10%
Black, Black British, Caribbean or African	4%	23%	19%	13%
Mixed, Other or Multiple Ethnic Group	5%	10%	9%	12%
White or White British	82%	59%	59%	65%
Unknown	N/A	N/A	3%	N/A

^a^Categories are based on Office for National Statistics (ONS) guidelines

### Study design and participation timeline

Participants may be recruited from 8 weeks gestation to delivery (Fig. 1). The visit and follow-up schedule is illustrated in Table 2. Participants who wish to provide only a particular sample type or who do not wish to complete follow-up are not discouraged, as these can contribute to cross-sectional analyses.

**Table 2:** INSIGHT-2 study schedule

	Timepoints						
	Pregnancy					Delivery	Childhood
	8–13^+6^ weeks	14–15^+6^ weeks	16–19^+6^ weeks	20–24^+0^ weeks	> 24 weeks	Peripartum	1, 3, 12, 18 & 24 months
Recruitment and informed consent	x—participants can join the study at any stage in pregnancy						
Demographic data	x—obtained at time of recruitment						x
Clinical data	x	x	x	x	x	x	x
Lifestyle data	x			x			x
Stress and mental health data	x		x		x		
Dietary recall	x		x		x		
Cervical measurements	x	x	x	x	x	x	
Cervicovaginal swabs	x	x	x	x	x		
Maternal blood	x	x	x	x	x	x	
Umbilical cord blood						x	
Neonatal blood						x	
Child blood							x
Placenta						x	
Placental image						x	
Maternal hair	x		x		x		
Maternal saliva^a^	x		x		x		
Child saliva^a^							x
Amniotic fluid	x—obtained when collection is clinically relevant						

^a^Future substudy, subject to funding

The maternal biological samples collected include cervicovaginal fluid, blood, and hair. Cord blood and placental samples are collected at birth, and child blood samples are collected at intervals ranging from 1 to 24 months of age. Saliva collection is possible in a future substudy. A description of the biological samples and the collection and storage details can be found under Additional file 1.

The data collected includes routine clinical information, mental health questionnaires, lifestyle information (sleep, exercise, and diet) (Table 3), fetal magnetic resonance imaging (MRI), and data linkages of participants and their children through the eLIXIR, Born in South London programme [35] (REC No: 23/SC/0116). The eLIXIR data-linkage project collects routine maternity and neonatal clinical patient data (uses opt-out consent at two NHS Foundation Trusts, GSTT and King's College Hospital NHS Foundation Trust (KCH)), mental health data, and primary care data.

**Table 3:** INSIGHT-2 data collection

Data type	Description	Relevance
Demographic	• Age • Ethnicity • Education level • Occupation • Lower layer Super Output Area (LSOA)	Demographic factors significantly influence pregnancy outcomes [53], and accounting for these factors is essential for effective monitoring, research, and interventions to improve maternal and child health, understanding disparities in pregnancy outcomes and targeting interventions to high-risk groups.
Clinical	• Preexisting medical history • Social history • Vaccinations during preconception months • Obstetric history • Family history of pregnancy complications • Height, weight and body mass index (BMI) • Use of medication during pregnancy • Delivery details • Complications of pregnancy • Neonatal outcome	Clinical data collection enables identification of risk factors, understanding of pregnancy complications’ aetiology, and development of better management strategies.
Lifestyle	• Smoking and alcohol consumption • Physical activity routine • Sleeping routine • Dietary regime These are recorded twice, in relation to 3 months prior to conception, and pregnancy period.	Lifestyle choices, such as diet, physical activity, smoking, and alcohol consumption are important modifiable risk factors which matter to patients andsignificantly impact pregnancy outcomes [54]. Adverse lifestyle habits have been shown to increase the risk of complications like gestational diabetes, hypertension, and preeclampsia [55, 56].
Stress and mental health	• Patient Health Questionnaire-9 (PHQ-9) [57] • Generalised Anxiety Disorder –7 (GAD-7) [58] • Perceived Stress Scale (PSS) [59] • Pregnancy Composite Abuse Scale (CAS) [60] • Psychosocial Index [61]	Preexisting and pregnancy-associated mental health illness, stress, and intimate partner violence are associated with sPTB, low birthweight, and other obstetric and perinatal complications [62–64].
Dietary recall	Quality of diet is assessed using a high-level questionnaire adapted through Intake24 [65], a self-completed computerised 24-h dietary recall system.	Maternal diet before and during pregnancy is a determinant of fetal growth and development, as well as maternal and infant health outcomes [66].
Cervical measurements	• Cervical length • Cervical stiffness using the Pregnolia system	Cervical length measurement using transvaginal ultrasound scan is the gold standard investigation for sPTB prediction and collected routinely clinically [67]. Correlating these measurements with other potential predictive biomarkers, e.g., from CVF or blood, may help to improve prediction of sPTB. Cervical stiffness measurements using the Pregnolia system (CE marked) in women at high risk of sPTB can be used as an adjunct to routine cervical length measurement. It has been shown that cervical softening (detected with this system) occurs prior to changes in cervical length measurement and so we plan to assess this further in our population [68].

## Research processes

### Patient and public involvement and engagement (PPIE)

PPIE input was sought during the planning phases of the study. Surveys were distributed to gather insights on the acceptability of specific sample collection methods. A steering group has been established that includes PPIE members to oversee and monitor the progress of the study, contribute to identifying new research questions, review requests for sub-studies, and ensure ongoing collaboration and alignment with the needs and perspectives of the community.

### Study sites

The main recruitment site is GSTT, with a planned expansion for the PISA sub-study to KCH. Other sites will be funding dependent.

### Participant identification and eligibility criteria

Potential participants are identified through medical records, recruitment material (such as flyers and posters) displayed in antenatal settings, social media advertising, or face‒to-face encounters during antenatal visits.

Potential pregnant participants are eligible if they are booked at our hospital sites and are ≥ 16 years old and ineligible if they are unable to consent or have a known significant congenital, structural, or chromosomal fetal abnormality.

### Consent and confidentiality

Participants provide written informed consent. Data and samples are link-anonymised in accordance with the UK Data Protection Act 2018 and the EU GDPR. For compliance with data protection legislation, the NHS Confidentiality Code, and the National Data Opt-Out Policy, potential participants are screened against national data opt-out. Participants can optionally consent to link study data to future health, education and social care outcomes via their NHS number, which includes Hospital Episode statistics, the Office for National Statistics and other related health/education and social care databases obtained through collaboration with the eLIXIR programme (REC No: 23/SC/01116).

## Ongoing projects

### Biomarkers for prediction of sPTB

**Fig. 2:**
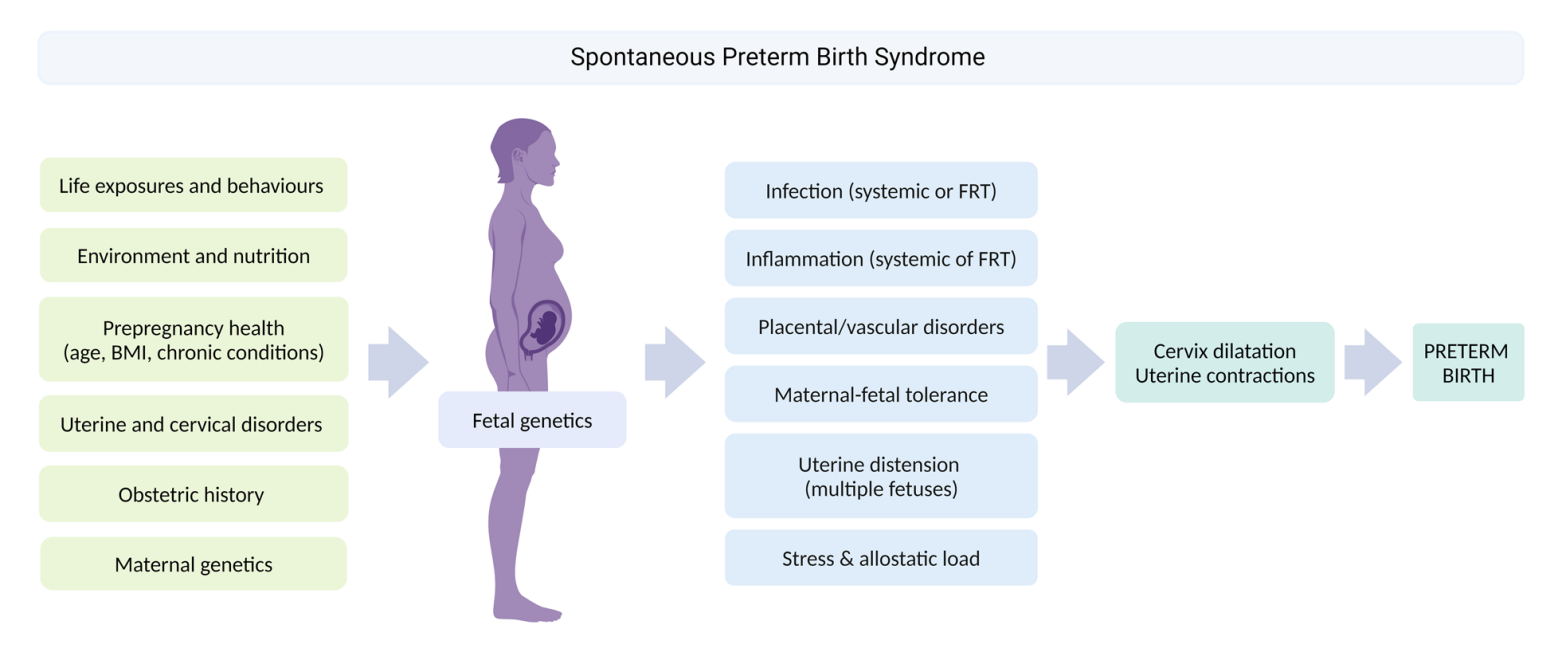
Spontaneous preterm birth syndrome. BMI: body mass index. FRT: female reproductive tract. Created in BioRender. Tribe, R. (2024) BioRender.com/o86z287

Current tools for prediction and potential prophylactic treatments to prevent sPTB are scarce [36]. sPTB is a complex syndrome with many biological pathways leading to a common presentation of premature activation of cervical shortening, with or without preterm premature rupture of fetal membranes (PPROM) and myometrial contractions (Fig. 2). The aim of this project is to characterise pregnant participants at moderate to high risk of sPTB to develop targeted approaches for risk prediction and treatment.

Participants are considered to be at risk of sPTB if they have a history of previous second trimester loss (≥ 16 weeks of gestation), PTB (≤ 37 weeks of gestation), PPROM (≤ 37 weeks of gestation), short cervical length (≤ 25 mm) on ultrasound at 18^+0^–24^+0^ weeks of gestation, any previous cervical procedure to treat abnormal smears (i.e., large loop excision, laser conisation, cold knife conisation or radical diathermy), multiple pregnancies, a history of full dilation-term caesarean section and known Müllerian abnormalities. Each risk factor may lead to a different pathophysiological mechanism of sPTB.

We aim to identify, test, and validate novel biomarkers in a range of biological samples via a range of approaches (described in the Sample analysis section) as well as the use of novel tools to assess cervical integrity. Machine learning and artificial intelligence (AI) approaches can be used to build algorithms using biomarker and phenotypic data to predict gestational age at birth and neonatal outcomes. The aim is to include these tools in existing clinical management tools such as the QUiPP app [37] and Tommy’s Clinical Decision Tool [38] to provide more personalised prediction and treatment.

The long-term consequences of preterm birth can be investigated through the longitudinal integration of biological samples and data from delivery and infancy, such as the presence of inflammatory cytokines in cord blood, histopathological assessment of the placenta to detect chorioamnionitis (linked to PTB), and developmental assessment of infants at key milestones.

Based on our previous studies, we anticipate that 200 pregnant people will be screened each year through the preterm surveillance clinic, of whom 50% (100/year) will take part. An estimated 500 participants at high risk of sPTB will be recruited over five years, with approximately 14% (≈70) delivering preterm (< 37 weeks gestation) and approximately 7% (≈35) delivering before 34 weeks gestation.

### Prenatal Drivers of Infant Islet Autoimmunity (PISA)

The PISA project aims to explore how maternal and fetal exposures during pregnancy influence the future risk of a child developing T1DM. Infants born to families with T1DM or born to mothers at risk of sPTB have an increased risk of developing this autoimmune disease when young [30, 31, 39]. Furthermore, a meta-analysis of eighteen studies [40] identified a role for maternal infection in increasing the risk of T1DM in children, with the strongest evidence pointing to enterovirus (EV) infection [41–44]. Moreover, pregnant women are likely to be exposed to EV infection, but whether infection during pregnancy can be protective and reduce the risk of T1DM is unknown.

First, maternal exposure to infections is monitored, as are environmental factors and their potential impact on the development of the immune system in infants. Maternal infection with enteroviruses is determined via a PCR-based screen. The profiles and functions of immune cells isolated from maternal, cord and child blood are analysed using flow cytometry, and multiplex ELISA is used for plasma analysis. Islet autoantibody testing will be performed at three time points during the babies’ first two years of life (at 12, 18 and 24 months of age). Follow-up data linkage allows for the detection of T1DM later in the child’s life.

Pregnancy complications and exposure to inflammation during pregnancy are associated with poor placentation and placental dysfunction, which can have long-term consequences for infant health [45, 46]. In recognition of the pivotal role of immune regulation by the placenta [47], placental immune cells are isolated [48], and their profile and function are analysed via flow cytometry. Understanding how exposure to adverse conditions, such as infection and maternal T1DM, affects the placenta during pregnancy contributes to a comprehensive understanding of placental immune cells in the context of T1DM.

Finally, fetal MRI scans are offered to those participants whose infants are considered at the highest risk of T1DM. These include those deemed at particularly high risk of sPTB and intrauterine infection [39, 49], who can be scanned as part of two linked studies (NANO REC No: 22/YH/0210, PRESTO REC No: 21/SS/0082), and those participants whose infants have a family history of T1DM under the MEERKAT study (REC No: 21/LO/0742). MRI scans allow for the assessment of fetal pancreas volume via T2-weighted images acquired in multiple orthogonal planes. These are subsequently reconstructed via a technique called deformable slice-to-volume reconstruction, which corrects for fetal motion [50]. As the pancreatic volume can be reduced by up to 26% in patients with T1DM within months of diagnosis, atrophy may begin years before the onset of clinical disease [51]. Indeed, reduced pancreatic function has been identified in infants prior to T1DM onset [52]. We hypothesise that a reduction in pancreatic volume may be a clinical marker of fetuses who could develop T1DM as infants.

### Data collection

As the importance of implementing core outcome sets for studies on pregnancy complications has been highlighted [69, 70], there is a growing need for comparable research data and harmonised protocols. In alignment with these guidelines, our study collects the suggested core data for investigations of pregnancy complications, ensuring comparability and compatibility across studies, while also gathering additional information to enrich the dataset and enhance the potential for comprehensive analyses and meaningful meta-analyses (see Table 3). Our database data structure is available upon request for those who wish to align with our collection strategy.

### Sample analysis

A variety of proteomic approaches are used to determine biomarker and stress and environmental marker concentrations, localisation and function (e.g., sandwich ELISA multiplex bead-based assays, nuclear magnetic resonance, mass spectrometry, and novel aptamer arrays; immunocytochemistry, flow cytometry, and exosome isolation, cargo analysis, and function testing in vitro and in vivo). Swabs (fluid/cells) and blood taken for biomarker and microbiome studies are also analysed via PCR/molecular/microarray techniques (e.g., RNA, noncoding RNA, metagenomic sequencing, lipidomics, metabolomics, epigenetics and single-cell/single nuclear RNA sequencing). Plasma/serum and peripheral blood mononuclear cells (PBMCs) are isolated for analysis and/or storage for future analysis, e.g., multiplex assays for cytokine profiling, other biomarker profiling, immune cell population analysis, and DNA and viral protein analysis. Plasma isolated from infant blood draws and home sampling kits is analysed for autoantibodies. The established genomic approaches will be used for genetic analyses (e.g., SNP polymorphism identification; human DNA sequencing).

### Statistical analysis

A formal data analysis plan will be drawn for each specific sub-study. Results will be analysed at each time point in a cross-sectional analysis, and on a case–control basis. For biomarker prediction studies, the overall usefulness of each marker for prediction of the primary endpoint will be expressed as a receiver operating characteristic area (ROC), with 95% confidence interval and p-value. The performance of the most useful markers will be described in terms of sensitivity, specificity and related measures for selected cut-points. Logistic regression will be used to identify possible combinations of markers; repeated measurements will be used to describe the change in test performance with gestation. A range of bioinformatic approaches will be used to integrate biological and clinical data to gain insight into risk factors, mechanisms, prediction, prevention, treatment and future health, education and social care outcomes.

## Discussion

### Expected outcomes

The long-term goal of this research is to leverage the identification of new biomarkers to aid early pregnancy prediction of common pregnancy complications and personalise treatment strategies that feed into the QUiPP App, Tommy’s Clinical Decision Tool, and future national (e.g., Saving Lives at Birth, NICE) and international guidelines. There is a need, for example, to find adjunct biomarkers for prediction of sPTB alternatives that can align with mechanistic causes.

The extensive collection of longitudinal samples and data in the INSIGHT-2 study enables both present investigations into the biological mechanisms of pregnancy complications and the establishment of a biobank to support future research and exploration of new hypotheses as scientific knowledge and technologies advance. This flexible approach to sample collection and analysis enhances its applicability across diverse hospitals and research settings. By gathering a wide range of biological samples, this study has the potential to accommodate new sites with varying capacities, ensuring broader participation.

To effectively manage the complexities of this multiyear research study, we adopted a structured approach to project management. We have a dedicated research study manager who is responsible for overseeing the various phases of the study, ensuring that all activities are coordinated, timelines are met, and resources are efficiently utilised. The success of this study is underpinned by the collaborative efforts of academic researchers, research midwives and practitioners, the bioresource management team, the research governance team, and the clinical team, each contributing their specialised expertise to ensure the study's integrity and progress.

Additionally, our study design includes a process for ongoing refinement through amendments. This iterative approach allows us to adapt to emerging data, address unforeseen challenges, and incorporate new methodologies or technologies as they become available. By maintaining flexibility and responsiveness, the study can ensure rigor, relevance, and the production of high-quality data throughout its duration.

Our diverse population allows us to capture a wide range of pregnancy complications and outcomes, which can vary significantly across different ethnic groups. This variability can provide a more comprehensive understanding of how these complications impact maternal and child health, enabling us to identify specific needs and tailor interventions for women from diverse backgrounds.

Moreover, including women from various ethnic groups ensures that our findings are generalisable. This approach enhances the external validity of our study, ensuring that our conclusions and recommendations are relevant and beneficial to the wider community. By focusing on a diverse population, we can also address health disparities and work towards reducing inequities in maternal and child health outcomes.

### Risk mitigation

To ensure the robustness and reliability of findings from INSIGHT-2, several risk mitigation strategies have been implemented. Recruitment strategies are designed to capture a diverse participant base, minimising selection bias and enhancing the generalisability of results. Comprehensive data management practices, including secure storage and regular audits, safeguard the integrity of the clinical and biological data collected. Potential confounding variables, such as preexisting medical conditions and socioeconomic factors, are systematically accounted for in data analysis to reduce their impact on study outcomes. One key risk is the potential loss of longitudinal data due to participant withdrawal or transfer of care. To address this, pathways have been established to request follow-up data from new care sites when participants transfer. To support retention, particularly during the follow-up visits in the child's early years, incentives such as gifts are provided to encourage continued participation. Additionally, not all participants may provide the necessary samples for each category of investigation; to mitigate this, over-recruitment strategies have been implemented to ensure sufficient sample sizes for biomarker analysis. Finally, rigorous ethical protocols are followed to safeguard participant safety and informed consent, maintaining compliance with regulatory standards. By integrating these measures, the study aims to mitigate potential risks and uphold the validity and reliability of its findings.

### Future directions

It is anticipated that in addition to current plans to develop biomarkers, treatments and influence guidelines, the INSIGHT-2 cohort and bioresource will be useful for a range of wider collaborations. Securing longer term funding will allow the follow up of children beyond two years of age and facilitate studies on maternal health beyond pregnancy. We anticipate expanding to other sites and sharing protocols to ensure alignment of our datasets with other studies for future metanalyses.

## Study registration

IRAS: 326577, REC: 23/WS/0114, CAG: 23/CAG/0094; OSF registration https://osf.io/vh9pn.

## Supplementary Information

**Additional file 1. **

**Additional file 2. **

**Additional file 3.**

## Data Availability

The INSIGHT research team welcomes applications from researchers interested in using data linkage or biobank. Researchers interested in using the INSIGHT & INSIGHT-2 data linkage/biobank should complete the INSIGHT research application form (RAF) (Additional file 2). The application will be submitted to the INSIGHT oversight committee, which determines the scientific merit of the study and reviews any potential overlap with existing agreements.

## Abbreviations

AI: Artificial intelligence
BMI: Body mass index
CAG: Confidentiality Advisory Group
CAS: Composite Abuse Scale
CLIC: A mechanistic investigation into cervical length and inflammatory changes
DNA: Deoxyribonucleic acid
ELISA: Enzyme-linked immunosorbent assay
EV: Enterovirus
EU: European Union
fFN: Fetal fibronectin
FGR: Fetal growth restriction
FRT: Female reproductive tract
GAD-7: Generalised Anxiety Disorder-7
GDM: Gestational diabetes mellitus
GDPR: General Data Protection Regulation
GSTT: Guy's and St Thomas' NHS Foundation Trust
HCRW: Health and Care Research Wales
HRA: Health Research Authority
INSIGHT: Investigation into Biomarkers to Predict Preterm Birth
INSIGHT-2: Mechanistic Studies into Pregnancy Complications and their Impact on Maternal and Child Health
IRAS: Integrated Research Application System
KCH: King’s College Hospital London NHS Foundation Trust
KCL: King’s College London
LSOA: Lower layer Super Output Area
MRI: Magnetic Resonance Imaging
NHS: National Health Service
PBMC: Peripheral blood mononuclear cell
PCR: Polymerase chain reaction
PE: Preeclampsia
PHQ-9: Patient Health Questionnaire-9
PISA: Prenatal Drivers of Infant Islet Autoimmunity
PPIE: Patient & Public Involvement & Engagement
PPROM: Preterm premature rupture of membranes
PSS: Perceived Stress Scale
PTB: Preterm birth
RAF: Request application form
REC: Research Ethics Committee
RNA: Ribonucleic acid
ROC: Receiver operating characteristic
SLE: Systemic lupus erythematosus
SNP: Single-nucleotide polymorphism
sPTB: Spontaneous preterm birth
UK: United Kingdom
WoSRES: West of Scotland Research Ethics Service
