# Supplementary material for: INSIGHT-2: mechanistic studies into pregnancy complications and their impact on maternal and child health—study protocol
Source: Reprod Health. 2024 Nov 28;21:177. doi: 10.1186/s12978-024-01911-0 (PMC11605920; doi:10.1186/s12978-024-01911-0)
Supplement: Supplementary file 1 — Additional file 1. [file 12978_2024_1911_MOESM1_ESM.docx]

## Supplementary material 1

*INSIGHT-2 sample collection:*

| **Sample Type** | **Purpose** | **Collection** | **Storage** |
| --- | --- | --- | --- |
| **Cervicovaginal swabs** | Analysing vaginal microbiota helps assess the microbial environment, with abnormal flora, particularly bacterial vaginosis, being associated with increased preterm birth (PTB) risk (1). Fetal fibronectin (fFN) is a protein that helps keep the amniotic sac attached to the uterine lining. Testing vaginal swabs for fFN is a valuable tool, as its presence in vaginal fluid between 22-34 weeks of pregnancy can indicate an increased risk of preterm birth (2). Additionally, the cervicovaginal fluid proteome reflects biochemical changes occurring in the vagina, cervix, and fetal membranes during pregnancy (3,4). Analysing this fluid can provide insights into cervical ripening and other physical changes that precede labour, both at term and preterm. | The speculum is inserted with a pea-sized amount of lubricating jelly (amount is minimised to avoid contamination of the swab). The low vaginal swab is taken 2-3 cm from the introitus and placed in 2 ml of PBS. The high vaginal swab is inserted into the posterior fornix of the vagina and rotated 360 degrees for 5 seconds before being placed in 2 ml of phosphate buffered saline (PBS). Without removing the speculum, the procedure is repeated with two microbiology swabs. One is smeared on glass slide then placed in 1 ml of liquid Amies. The other one is placed in 2 ml of TE buffer. Both these tubes are agitated for 30 seconds than swabs are thrown away. | Cervicovaginal fluid and cell pellets are isolated within 1 hour of collection and stored at -80°C. Aliquots are reserved for biobank purposes and for future assessments. Amies, TE buffer and microbiology slide are frozen at -80°C. |
| **Maternal blood** | Blood allows for investigation of circulating factors that may influence pregnancy health and the development of pregnancy complications (e.g., multiplex assays for cytokine profiling, biomarker profiling, immune cell population analysis, DNA, viral protein analysis, etc.). As this is being collected prospectively, other biological measurements using blood or its components may be made as discoveries are reported in the literature. | Up to 20 ml of blood are collected via venepuncture into blood collection tubes for the isolation of plasma, serum and peripheral blood mononuclear cells (PBMCs). The residual red cells and buffy coat (for DNA) are also be isolated for future analysis. Finally, up to 5 ml of the collected blood can be stored as whole blood or used for the isolation of different types of white blood cells. | PBMCs and CBMCs are stored in liquid nitrogen. Plasma, serum, whole blood and buffy coats are stored at -80°C. Aliquots are reserved for biobank purposes and for future assessments.  The child samples at 12, 18 and 24 months are also analysed by an external laboratory for presence of islet autoantibodies. |
| **Umbilical cord blood** | Umbilical cord blood and child blood is be used to measure components of the immune system, including immune cells and inflammatory signals, and explore mechanisms which lead to health issues in infancy and childhood. Additionally, child blood samples collected at 12, 18 and 24 months is be used for autoantibody testing (biomarker of risk of child developing autoimmune disease). | After delivery, the umbilical cord is clamped, and the cord blood is be collected. A 3 cm sample of the umbilical cord is collected and undergoes flash freezing in liquid nitrogen. The cord blood is then be processed to isolate the cord blood mononuclear cells (CBMCs) and plasma. |  |
| **Child blood** |  | Child blood samples are taken at 1 month, 3 month, 12 months and 24 months. A 1.3 ml sample is collected by venepuncture. At 18 months, a home blood pack is sent for participants to collect to collect 60 µL by finger prick. |  |
| **Placenta** | Pregnancy complications and exposure to inflammation during pregnancy are associated with poor placentation and placental dysfunction, which can have long-term consequences on infant health (5,6). | A photograph of the untrimmed placenta is taken using a digital camera positioned in a fixed location, with rulers placed to record tissue dimensions. Subsequently, the placenta is trimmed and weighed. Eight representative placental tissue samples are collected randomly, comprising four centrally located and four peripherally located samples. Each of the central and peripheral samples are paired and stored together in four separate tubes. | The first tube undergoes flash freezing in liquid nitrogen for protein studies. The second tube is stored in RNA later overnight and then frozen at -80°C for gene expression analysis. The third tube containing the samples is processed with formalin to create paraffin wax-embedded tissue blocks for morphological examination and protein immuno-localisation studies. The fourth sample selection undergoes processing to isolate immune cells, resulting in a single-cell suspension that is subjected to flow cytometry analysis. |
| **Maternal hair** | Hair cortisol concentrations have been associated with psychological distress in pregnancy (7). Less well-established biomarkers of interest include brain-derived neurotrophic factor and endocannabinoids (8). | A 1 cm-thick full-length hair sample is cut close to the scalp from the posterior vertex. | The hair sample is stored in aluminium foil at room temperature until analysis. |
| **Maternal & child saliva** | The measurement of salivary progesterone shows promise as a predictor for preterm birth, with high acceptability among pregnant women (9,10). Additionally, salivary samples represent a valuable alternative route to hair for cortisol extraction, for assessing stress levels and other physiological conditions (11,12).  This is optional as will be collected for discrete sub study only. | Participants are provided with a universal specimen container to take home. They take the sample immediately upon waking after rinsing their mouth with water. They are then asked to keep the sample cold until it has been delivered to the study team. Participants are asked to collect the sample on a day where they have a preexisting appointment. Infant saliva is collected through a specially designed buccal swab. | Sent for analysis directly (salivary cortisol protocol how do they do it) and cortisol measured automatically. |
| **Amniotic fluid** | Acute inflammation of the placenta and umbilical cord, known as chorioamnionitis and funisitis, respectively, contributes to 40% of preterm births, often attributed to intra-amniotic infection (13,14). Diagnosis of these conditions remains clinically challenging, primarily relying on invasive amniotic fluid culturing. However, exploring correlations between amniotic fluid biomarkers and those in cervicovaginal fluid could enhance understanding of preterm birth mechanisms and aid in developing predictive tests for chorioamnionitis. | 15-20 ml of amniotic fluid is only collected if amniocentesis is done for a clinical indication, following the Royal College of Obstetricians and Gynaecologists guidance (15). | The sample is collected on ice, aliquoted and snap frozen. |

## References

1. Hoffmann E, Váncsa S, Váradi A, Hegyi P, Nagy R, Hamar B, et al. Routine screening of abnormal vaginal flora during pregnancy reduces the odds of preterm birth: a systematic review and meta-analysis. Sci Rep. 2023 Aug 25;13(1):13897.

2. Leitich H, Egarter C, Kaider A, Hohlagschwandtner M, Berghammer P, Husslein P. Cervicovaginal fetal fibronectin as a marker for preterm delivery: a meta-analysis. Am J Obstet Gynecol. 1999 May;180(5):1169–76.

3. Zegels G, Van Raemdonck GA, Tjalma WA, Van Ostade XW. Use of cervicovaginal fluid for the identification of biomarkers for pathologies of the female genital tract. Proteome Sci. 2010 Dec 8;8(1):63.

4. Heng YJ, Liong S, Permezel M, Rice GE, Di Quinzio MKW, Georgiou HM. Human cervicovaginal fluid biomarkers to predict term and preterm labor. Front Physiol. 2015 May 13;6:151.

5. Couture C, Brien ME, Boufaied I, Duval C, Soglio DD, Enninga EAL, et al. Proinflammatory changes in the maternal circulation, maternal–fetal interface, and placental transcriptome in preterm birth. Am J Obstet Gynecol. 2023 Mar 1;228(3):332.e1-332.e17.

6. Musa E, Salazar-Petres E, Arowolo A, Levitt N, Matjila M, Sferruzzi-Perri AN. Obesity and gestational diabetes independently and collectively induce specific effects on placental structure, inflammation and endocrine function in a cohort of South African women. J Physiol. 2023;601(7):1287–306.

7. Khoury JE, Giles L, Kaur H, Johnson D, Gonzalez A, Atkinson L. Associations between psychological distress and hair cortisol during pregnancy and the early postpartum: A meta-analysis. Psychoneuroendocrinology. 2023 Jan;147:105969.

8. Koenig AM, Gao W, Umlauft M, Schury K, Reister F, Kirschbaum C, et al. Altered hair endocannabinoid levels in mothers with childhood maltreatment and their newborns. Biol Psychol. 2018 May 1;135:93–101.

9. PL.55 Testing Saliva for the Prediction of Preterm Birth: How acceptable is this method to women at risk? | ADC Fetal & Neonatal Edition [Internet]. [cited 2023 Dec 5]. Available from: https://fn.bmj.com/content/98/Suppl_1/A69.3

10. Sharma P, Khan S, Ghule M, Shivkumar VB, Dargan R, Seed PT, et al. Rationale & design of the PROMISES study: a prospective assessment and validation study of salivary progesterone as a test for preterm birth in pregnant women from rural India. Reprod Health. 2018 Dec 22;15:215.

11. Cruickshank T, Porter T, Laws SM, Ziman M, Bartlett DM. Hair and salivary cortisol and their relationship with lifestyle, mood and cognitive outcomes in premanifest Huntington’s disease. Sci Rep. 2021 Mar 9;11(1):5464.

12. Gonzalez D, Jacobsen D, Ibar C, Pavan C, Monti J, Fernandez Machulsky N, et al. Hair cortisol measurement by an automated method. Sci Rep. 2019 Jun 3;9(1):8213.

13. Jain VG, Willis KA, Jobe A, Ambalavanan N. Chorioamnionitis and neonatal outcomes. Pediatr Res. 2022 Jan;91(2):289–96.

14. Galinsky R, Polglase GR, Hooper SB, Black MJ, Moss TJM. The consequences of chorioamnionitis: preterm birth and effects on development. J Pregnancy. 2013;2013:412831.

15. Navaratnam K, Alfirevic Z, the Royal College of Obstetricians and Gynaecologists. Amniocentesis and chorionic villus sampling. BJOG Int J Obstet Gynaecol. 2022;129(1):e1–15.
