# Supplementary material for: INSIGHT-2: mechanistic studies into pregnancy complications and their impact on maternal and child health—study protocol
Source: Reprod Health. 2024 Nov 28;21:177. doi: 10.1186/s12978-024-01911-0 (PMC11605920; doi:10.1186/s12978-024-01911-0)
Supplement: Supplementary file 2 — Additional file 2. [file 12978_2024_1911_MOESM2_ESM.docx]

**INSIGHT & INSIGHT-2**

**Data and Samples Request Application Form**

## Directions

The principal applicant responsible for overseeing the project and controlling the laboratory and personnel who will receive, use and process the requested specimens should complete this application. The CI of INSIGHT/INSIGHT-2 would normally be a co-applicant and other relevant PI’s included on a case-by-case basis. The wider INSIGHT/INSIGHT-2 collaborative group should be acknowledged on publications.

When submitting a request for data and/or samples, for use in more than one study, please complete separate copies of the Request Application Form.

If approved, the data and samples will be allocated for your study for 1 year, with project activation normally anticipated within 6 months. If after one year, no progress is reported, they will be made available for other researchers to request. The analysed data will be reviewed by the INSIGHT Research Team before being submitted for publications.

For academic projects there will be a component of cost recovery and a handling charge for retrieving data and samples to cover the administrative costs (commercial applications will require full cost recovery). Applicants are responsible for covering these costs and all charges that arise for shipping the samples. The courier used for transferring samples must be approved by the committee.

Please submit the application via email to [insight-2@kcl.ac.uk](mailto:insight-2@kcl.ac.uk)

## Applicants

**Principal applicant:**

| Name: |  |
| --- | --- |
| Organisation: |  |
| Email: |  |
| Telephone: |  |
| Address: |  |

**Co-applicants:**

| Name: | Role: | Institution/Organisation: |
| --- | --- | --- |
|  |  |  |
|  |  |  |
|  |  |  |
|  |  |  |
|  |  |  |
|  |  |  |
|  |  |  |
|  |  |  |

## Project information

| Title: |  |
| --- | --- |
| Planned start date: |  |
| Planned end date: |  |
| Project category: | Research  Audit*  Service Development  Service Evaluation  Quality Improvement  Implementation Science  Other  **Please confirm the project has received appropriate clinical governance approval and email the approval to the INSIGHT-2 research office along with this application* |

Does the project have ethics under INSIGHT-2?

Yes  No *(– if not, specify ethics details below including REC reference number)*

|  |
| --- |

Does the project require recontacting the selected participants? Yes  No

## Collaboration type

Does the proposed project have Commercial Partnerships?Yes  No

If yes, please provide contact name and details:

|  |
| --- |

Is Intellectual Property (IP) likely to arise from this research?Yes  No

If yes, please provide further details and the contact details of the IP officer for your Institution:

|  |
| --- |

## Funding

Is funding required to complete the project?Yes  No *(– please move to section 5)*

| Funding Status | Yes | No | Funder/Amount funded/Date |
| --- | --- | --- | --- |
| Planning to apply for funding |  |  |  |
| Applied for funding |  |  |  |
| Outcome of funding application expected date |  |  |  |
| Funded |  |  |  |

## Justification *(no more than 2 pages)*

| Outline the background of the study:  *(If a grant has already been approved for this project, please attach the grant application.)* |
| --- |
|  |
| Outline the hypothesis of the study: |
|  |
| Outline study aims and the sample size calculation: |
| Primary objective  Secondary objectives  Sample size calculation |
| References |
|  |

## Participant type needed

| Specifics | Gestational age range/Infant age range | Total #Subjects | Total #Controls |
| --- | --- | --- | --- |
| Normal pregnancy |  |  |  |
| Pregnancy complications |  |  |  |
| Fetal |  |  |  |
| Newborn/Child |  |  |  |

| Maternal Age: | Any | Specific ages, list: | |
| --- | --- | --- | --- |
| Fetal Gender: | Any | Female Only | Male Only |
| Pregnancy Outcome: | Term  Preterm  Stillbirth  Fetal Growth Restriction  Preeclampsia  N/A  Other: | | |
| Child Health Outcome: | Healthy  T1D  Other: | | |

## Samples request

| Pregnant women samples | | |
| --- | --- | --- |
| Specimen Type | **Number of aliquots required per participant** | **Minimum volume required for assay** |
| Serum |  |  |
| EDTA Plasma |  |  |
| Whole blood |  |  |
| Ficoll-diluted plasma |  |  |
| PBMCs |  |  |
| EDTA Buffy coat |  |  |
| Microbiology swab – Liquid Amies |  |  |
| Microbiology swab – Slide |  |  |
| Microbiology swab – TE Buffer |  |  |
| Saliva (hormones) |  |  |
| Saliva (microbiology) |  |  |
| Saliva (hormones) |  |  |
| Cytobrushing fluid |  |  |
| Cell pellet (CBS) |  |  |
| High vaginal swab |  |  |
| Cell pellet (HVS) |  |  |
| Low vaginal swab |  |  |
| Cell pellet (LVS) |  |  |
| Amniotic fluid |  |  |

| Placental Samples | | |
| --- | --- | --- |
| Specimen Type | **Number of aliquots required per participant** | **Minimum volume required per assay** |
| Snap frozen |  |  |
| RNA Later |  |  |
| Paraffin slides |  |  |
| Cell Suspension |  |  |
| Pictures of placenta(s) – *if taken*: ☐ Yes ☐ No | | |

| Infant Samples | | |
| --- | --- | --- |
| Specimen Type | **Number of aliquots required per participant** | **Minimum volume required per assay** |
| Cord blood plasma |  |  |
| Cord blood PBMCs |  |  |
| Infant blood plasma |  |  |
| Infant blood PBMCs |  |  |

## Data request

| Clinical data |
| --- |
| Standard information provided with coded-linked specimens comprises of pregnancy outcome/clinical diagnosis, maternal age, gestational age, infant age and infant gender, if available. Select preference:  Standard information only;  Standard information and additional information indicated below;  No clinical data required.  Additional information from data collection forms may be available upon request. Indicate additional information required:  Stress, mental health and life experiences questionnaires  Dietary recall outcomes  First trimester antenatal visit  Second trimester antenatal visit  Third trimester antenatal visit  Delivery details  Please justify your data selection: |

## Agreement

**Signature:**

|  |
| --- |

If you are sending this form by email then you should note that in the absence of this signature, the email of this proposal constitutes your personal certification that the details are correct.

**Date:**

|  |
| --- |

**Name** (*on behalf of applicants)***:**

|  |
| --- |

## For internal use only (INSIGHT-2 Team)

**Allocated application number:**

|  |
| --- |

| Stage | Date | Outcome | Comments |
| --- | --- | --- | --- |
| Received application form |  | Completed  Yes  No |  |
| Discussed with Team |  | Declined  Declined but can resubmit  Provisionally accepted  Accepted |  |
| Re-submitted for review |  |  |  |
| Re-discussed with Team |  | Declined  Declined but can resubmit  Provisionally accepted  Accepted |  |
| Project agreement requested |  |  |  |
| All contracts/agreements confirmed |  |  |  |
| Data/samples shared |  |  |  |
