# Supplementary material for: INSIGHT-2: mechanistic studies into pregnancy complications and their impact on maternal and child health—study protocol
Source: Reprod Health. 2024 Nov 28;21:177. doi: 10.1186/s12978-024-01911-0 (PMC11605920; doi:10.1186/s12978-024-01911-0)
Supplement: Supplementary file 3 — Additional file 3. [file 12978_2024_1911_MOESM3_ESM.docx]

## Supplementary material 3

*INSIGHT-2 Team:*

1. **King’s College London & Guy’s and St Thomas’ NHS Foundation Trust:**

Abigail Adeosun, Clinical Research Practitioner; Amy Tiltman, Research Midwife; Andrew Shennan, Site Principal Investigator; Angela Chiapparino, Research Midwife; Anna Brockbank, Research Technician; Arianna Towler, Clinical Research Practitioner; Caitlin Giles, Research Assistant; Cally Gill, School Bioresource Manager; Carlotta Valensin, Research Study Manager; Coral Xing, Research Assistant; Daniela Pereira Carvalho, Research Associate; Debbie Finucane, Senior Research Midwife; Declan Symington, Research Midwife; Deena Gibbons, PISA Principal Investigator; Delphine Strub, Research Midwife; Emilie Côté, Clinical Research Fellow; Gemma Baillie, Research Midwife; Glen Nishku, Research Manager; Hayley Tarft, Senior Research Midwife; Hannah Rosen O’Sullivan, Clinical Research Fellow; Hilary Thompson, INSIGHT-2 Co-Lead Research Midwife; Hira Husain, Clinical Research Practitioner; Jenny Carter, Senior Research Midwife; Jo Bennett, Research Midwife; Julie Wade, Senior Research Midwife; Laura McCabe, Research Matron; Laura Van Der Krogt, Clinical Research Fellow; Megan Hall, Clinical Research Fellow; Molly Dillon, Clinical Research Practitioner; Naomi Moulla, Bioresource Technician; Natalie Suff, Clinical Lecturer; Nicole Moriarty, Clinical Research Fellow; Pamela Taylor-Harris, Technical Manager; Paul Seed, Trial Biostatistician; Rachael Gardner, INSIGHT-2 Co-Lead Research Midwife; Rachel Tribe, Chief Investigator; Soline Caprioli, Clinical Research Practitioner; Vicky Robinson, Senior Research Midwife.

1. **INSIGHT-2 Steering Group**

Anita Banerjee, Independent Member; Carlotta Valensin, Study Manager; Glen Nishku; Research Manager; Laura McCabe, Research Matron; Lisa Story, Independent Member; Rachel Tribe, Study CI; Aaron Fox, PPIE Independent Member; Claire Macdonald, PPIE Independent Member; Deena Gibbons, PISA Study Co-PI; Andrew Shennan, Site PI; Rachel Brown, PPIE Independent Member, Zoë Vowles, Independent Member.

1. **Collaborators on the PISA Study Grant**

Andrew Shennan, Professor of Obstetrics; Anita Banerjee, Obstetric Physician and Diabetes and Endocrinology Consultant; Anna Esther Long, Lecturer in Diabetes; Deena Gibbons, Professor in Early Life Immunology; Jana Hutter, Senior Research Fellow; Lisa Story, Senior Lecturer and Honorary Consultant in Obstetrics and Fetal Medicine; Rachel Tribe, Professor of Maternal and Perinatal Sciences; Rui Pedro Galão, Lecturer in Infectious Diseases; Timothy Tree, Professor of Immune Regulation and Immunotherapy.

1. **Team at King’s College Hospital NHS Foundation Trust**

Kate Hunt, Consultant in Diabetes and General Internal Medicine; Pearl Dulawan, Research Matron; Sophie Webster, Senior Clinical Research Midwife; Kate Bramham, Reader of Nephrology & Maternal Medicine and Honorary Consultant Nephrologist.
